# Metabolic engineering of indole pyruvic acid biosynthesis in *Escherichia coli* with *tdiD*

**DOI:** 10.1186/s12934-016-0620-6

**Published:** 2017-01-03

**Authors:** Yelin Zhu, Yan Hua, Biao Zhang, Lianhong Sun, Wenjie Li, Xin Kong, Jiong Hong

**Affiliations:** School of Life Sciences, University of Science and Technology of China, Hefei, Anhui 230027 People’s Republic of China

**Keywords:** Indole pyruvic acid, *tdiD*, Aminotransferase, *tnaA*

## Abstract

**Background:**

Indole pyruvic acid (IPA) is a versatile platform intermediate and building block for a number of high-value products in the pharmaceutical and food industries. It also has a wide range of applications, such as drugs for the nervous system, cosmetics, and luminophores. Chemical synthesis of IPA is a complicated and costly process. Moreover, through the biosynthesis route employing l-amino acid oxidase, the byproduct hydrogen peroxide leads the degradation of IPA. TdiD, identified as a specific tryptophan aminotransferase, could be an alternative solution for efficient IPA biosynthesis.

**Results:**

*Escherichia coli* strain W3110, which demonstrates basic production when supplied with tryptophan, was engineered for IPA biosynthesis. Several strategies were implemented to improve IPA production. First, through incorporating the codon-optimized *tdiD* into W3110, IPA levels increased from 41.54 ± 1.26 to 52.54 ± 2.08 mg/L. Second, after verifying the benefit of an increased phenylpyruvate pool, a YL03 strain was constructed based on a previously reported mutant strain of W3110 with a plasmid carrying *aroF* ^*fbr*^ and *pheA* ^*fbr*^ to further improve IPA production. The recombinant YL03 strain accumulated IPA at 158.85 ± 5.36 mg/L, which was 3.82-fold higher than that of the wild-type W3110 strain. Third, optimization of *tdiD* ^*co*^ expression was carried out by replacing the Trc promoter with a series of constitutively active promoters along with increasing the plasmid copy numbers. The highest IPA production was observed in YL08, which achieved 236.42 ± 17.66 mg/L and represented a greater than 5-fold increase as compared to W3110. Finally, the effects of deletion and overexpression of *tnaA* on IPA biosynthesis were evaluated. The removal of *tnaA* led to slightly reduced IPA levels, whereas the overexpression of *tnaA* resulted in a considerable decline in production.

**Conclusions:**

This study illustrates the feasibility of IPA biosynthesis in *E. coli* through *tdiD*. An efficient IPA producing strain, YL08, was developed, which provides a new possibility for biosynthesis of IPA. Although the final production was limited, this study demonstrates a convenient method of IPA synthesis.

**Electronic supplementary material:**

The online version of this article (doi:10.1186/s12934-016-0620-6) contains supplementary material, which is available to authorized users.

## Background

**Fig. 1:**
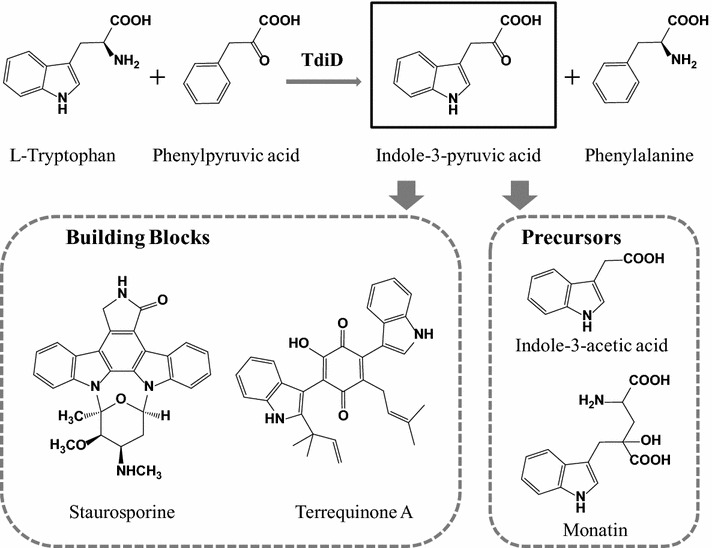
IPA synthesis reaction catalyzed by TdiD and IPA applications

Indole pyruvic acid (IPA) is well known as the first step product in the IPA-pathway of the plant hormone indole-3-acetic acid (IAA) biosynthesis [1]. IPA is also used as the precursor of the sweetener monatin [2] (Fig. 1). Chromopyrrolic acid can be produced from IPA directly, thereby generating indolocarbazole compounds with antitumor activities, such as staurosporine [3] (Fig. 1) and rebeccamycin [4]. Furthermore, IPA constitutes the fundamental building block of bis-indolylquinones, such as terrequinone A [5] (Fig. 1), semicochliodinol, and hinnuliquinone [6]. In recent decades, owing to their antiretroviral, anti-diabetes and cytotoxic bioactivities, these compounds have garnered significant attention [6].

Moreover, IPA itself is of high pharmaceutical importance. IPA has analgesic and sedative properties, particularly, its analgesic action does not induce drug resistance [7]. For anxious people, IPA is a safe drug without withdrawal effects, and can help people release stress as well as generate feelings of relaxation and happiness [8]. Besides, IPA counteracts endocrine improvement during stressful situations [9]. After administration, serotonin and melatonin are the most prominent products of IPA, which have positive effects on insomnia [10]. As a neuronal protecting agent [11], IPA maintains its antioxidant function to inhibit radical damage, and thus protects the brain from pathological impairment during aging [12]. In addition, IPA is patented as a cosmetic agent for sun protection and anti-aging [13]. And it could be a potential source of luminophores due to the characteristics of its chemiluminescence spectrum [14].

The chemical methods for IPA synthesis use either indole or tryptophan (Trp) as the initial raw material, but the procedures are complicated and costly [15, 16]. Biosynthesis, on the other hand, has a lot of advantages. Employing an l-amino acid oxidase or aminotransferase, *Escherichia coli* was able to successfully produce IPA from L-Trp [17, 18]. For the l-amino acid oxidase catalytic reaction, the synthesis of IPA is accompanied by the generation of hydrogen peroxide in equivalent mole ratio to IPA. Hydrogen peroxide can induce IPA degradation. Therefore, catalase activity either from the inducible and endogenous *E. coli* catalase [19] or exogenous catalase expression is required, in order to ensure IPA protection from the degradation mediated by hydrogen peroxide. Nevertheless, the remaining hydrogen peroxide can lead to oxidative stress [20, 21] as well as the undesired oxidation of further product. Therefore, elaborate regulation of catalase expression is necessary. However, through amino acid aminotransferase (AAT), the other product in addition to IPA would be an amino acid corresponding to the amino acceptor. Unlike the IPA biosynthesis process catalyzed by amino acid oxidase, no further steps were needed to remove the toxic byproduct and modulate redox balance when an AAT is utilized. Moreover, the accumulation of an amino acid as the other product, which is of great commercial value [22], provides an extra economic benefit. Therefore, in order to develop a universally applicable platform to improve IPA production and facilitate the biosynthesis of the follow-up sophisticated compound, utilization of an AAT is the optimal approach.

There are several AATs in *E. coli*. Similar to aspartate aminotransferase (AspC) and aromatic amino acid aminotransferase (TyrB), almost all of these enzymes are multispecific [23], and responsible for the synthesis of corresponding amino acids. In order to construct an IPA biosynthetic pathway with concise route, which can be accurately controlled without concerns for substrate preference, the utilization of an AAT with substrate specificity for Trp is desirable. Recently, with the elucidation of the terrequinone A biosynthesis pathway [5], *tdiD* derived from *Aspergillus nidulans* was characterized as an l-tryptophan:phenylpyruvate aminotransferase [6] (Fig. 1). This finding encourages the exploration of a new approach for IPA biosynthesis with TdiD in which only one catalysis step is needed to produce IPA from Trp. In this study, we demonstrate the construction of a new pathway for IPA production. The IPA producing pathway was established through recombinant expression of codon-optimized *tdiD* (*tdiD* ^*co*^). IPA production was subsequently improved by increasing the substrate availability, blocking the branch pathway, and optimizing *tdiD* ^*co*^ expression. The influence of *tnaA* was also investigated in detail. Finally, an effective IPA production strain that can be engineered for further bioactive compound synthesis was developed. The procedure reported here represents a new vision into IPA biosynthesis and provides valuable perspective for this biological route.

## Results and discussion

### The incorporation of *tdiD* into *E. coli* W3110

TdiD catalyzes the transamination of L-Trp to form IPA. The two substrates, L-Trp and phenylpyruvate (PPA), are downstream metabolites of the shikimate pathway in *E. coli*. In addition to IPA, the other product is phenylalanine (Phe) which has extensive applications, functioning as a nutraceutical as well as the precursor for the generation of food additives and pharmaceutically active compounds [22]. Here, we managed to improve the production of IPA using the transaminase TdiD supplemented with 2 g/L Trp.

**Fig. 2:**
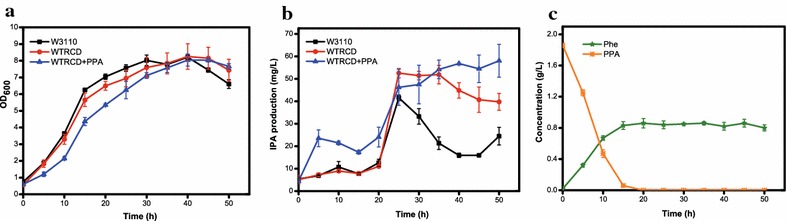
Culture profiles of W3110, WTRCD and WTRCD + PPA. **a** Growth curve; **b** IPA production curve; **c** Phe and PPA concentrations of WTRCD in PPA feeding experiment

*tdiD* ^*co*^ under the control of the Trc promoter was inserted into the medium-copy-number pET24b and named pTRCD (Table 1). Thus, *E. coli* strain WTRCD (Table 1) transformed pTRCD exhibited moderate TdiD expression. There was no distinct difference between W3110 and WTRCD in cell growth (Fig. 2a), indicating no detrimental effects caused by the existence of pTRCD. The WTRCD strain displayed enhanced IPA levels compared to the W3110 strain. Generally, the IPA production of W3110 and WTRCD increased continuously within 0–25 h, and maxed after 25 h of cultivation (the 0 h of cultivation started at the point of IPTG induction). At 25 h, the WTRCD strain produced a final titer of 52.54 ± 2.08 mg/L IPA, which is 26.48% more than the IPA produced in the W3110 strain (41.54 ± 1.26 mg/L) (Fig. 2b). After 25 h, IPA levels started to decrease in both strains, which can be ascribed to the decomposition [26]. However, the IPA production of W3110 strain declined sharply while the IPA production in WTRCD strain decreased gradually (Fig. 2b). W3110 demonstrates basic IPA production due to the inherent multispecific AATs, which could convert Trp into IPA when supplied with Trp. AspC and TyrB, which have been used for IPA synthesis as an intermediate step in previous reports [1, 18], were considered as the main enzymes for the IPA basic production in W3110 strain. And this assumption has been verified by the negligible IPA production in the *aspC* and *tyrB* mutants strain which is indicated in the following section (Fig. 4b). AspC is in favor of the reverse reaction converting IPA to Trp [27, 28]. On the other hand, though TyrB catalyzes the reaction with a much lower *K*m than AspC [29], its expression is severely repressed by the products [30, 31]. Therefore, after 25 h cultivation, IPA biosynthesis in W3110 were repressed while the consumption and degradation of IPA continued, resulting in the dramatically reduced IPA concentration.

**Table 1:** Strains and plasmids used in this study

	Genotype/description	Source/references
Strain		
DH5α	*lacZ*ΔM15 *endA1 recA1 relA1 gyrA96 deoR nupG λ* ^−^	Transgene Bio
W3110	F^−^ *λ* ^−^ *rph*-*1 INV (rrnD, rrnE)*	[24]
WTRCD	W3110 with pTRCD	This study
Sun21	W3110 Δ *tyrB*::FRT, Δ*aspC*::FRT, *tyrA16*::Tn10	[24]
Zhu01	W3110 Δ*tyrB*, Δ *aspC*, Δ*tnaA*, *tyrA16*::Tn10	This study
YL01	Sun21 with pTRCD	This study
YL02	Sun21 with pSUFAQ	This study
YL03	Sun21 with pTRCD and pSUFAQ	This study
YL04	Sun21 with p12D and pSUFAQ	This study
YL05	Sun21 with p14D and pSUFAQ	This study
YL06	Sun21 with p16D and pSUFAQ	This study
YL07	Sun21 with p18D and pSUFAQ	This study
YL08	Sun21 with p20D and pSUFAQ	This study
YL09	Sun21 with pDRD and pSUFAQ	This study
YL10	Sun21 with pMOD and pSUFAQ	This study
YL11	Sun21 with pDMD and pSUFAQ	This study
YL12	Zhu01 with p20D and pSUFAQ	This study
YL13	Sun21 with pTAD and pSUFAQ	This study
YL14	Zhu01 with pTAD and pSUFAQ	This study
Plasmid		
pET24b-*tdiD*	*Nde*I/*Not*I (*tdiD*)	[5]
pSUFAQ	pSU2718 derivative, *aroF* ^*fbr*^, *pheA* ^*fbr*^, *lacI* ^*q*^	[24]
pBJEI-6409	Codon-optimized genes of MEV pathway, Trc promoter	Addgene
pUC57-D	Synthetic *tdiD* ^*co*^	Sangon
pUC57-18	Synthetic OXB18 promoter	Sangon
pT7D	pET24b-*tdiD* derivative, T7 promoter, *tdiD* ^*co*^	This study
pTRCD	pT7D derivative, Trc promoter	This study
p12D	pTRCD derivative, OXB12 promoter	This study
p14D	pTRCD derivative, OXB14 promoter	This study
p16D	pTRCD derivative, OXB16 promoter	This study
p18D	pTRCD derivative, OXB18 promoter	This study
p20D	pTRCD derivative, OXB20 promoter	This study
pDRD	p20D derivative, Rom coding sequence deleted	This study
pMOD	p20D derivative, origin mutation-108A(108C)	This study
pDMD	pMOD derivative, Rom coding sequence deleted	This study
pTAD	p20D derivative, *tnaA* expression cassette	This study
pKD13	*bla* FRT-*kan* -FRT	[25]
pKD46	*bla αβ exo* (red recombinase), temperature conditional replicon	[25]
pCP20	*bla cat*, temperature sensitive replicon, temperature inducible FLP recombinase	[25]

### PPA feeding experiment

**Fig. 3:**
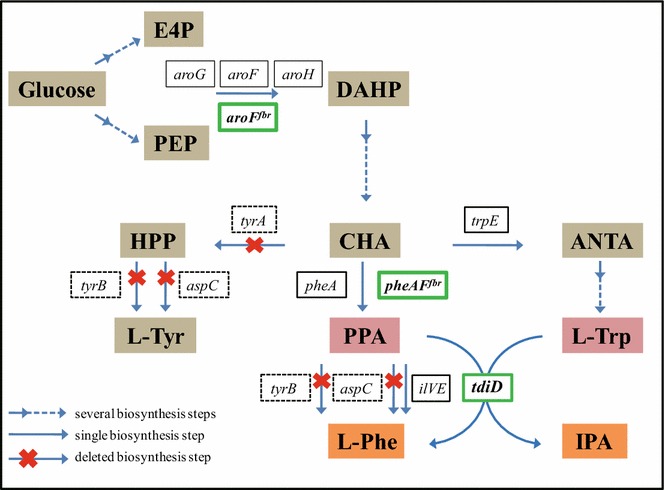
Metabolic engineering for IPA biosynthesis in *E. coli.* Genes of the catalytic enzyme are stated in the *boxes*. *Boxes with solid lines* represent *E*. *coli* inherent genes, *boxes with dashed lines* represent knockout genes, and *boxes with bold lines* represent heterogeneous genes expressed in plasmids. *ANTA* anthranilate, *CHA* chorismate, *E4P* erythrose-4-phosphate, *DAHP* 3-deoxy-d-arabino-heptulosonate-7-phosphate, *HPP* 4-hydroxyphenylpyruvate, *IPA* indole pyruvic acid, *L-Phe* l-phenylalanine, *L-Trp* l-tryptophan, *L-Tyr* l-tyrosine, *PEP* phosphoenolpyruvate, *PPA* phenylpyruvate. Enzymes coded by genes: *aroF*, DAHP synthase; *aroG*, DAHP synthase; *aroH*, DAHP synthase; *aroF* ^*fbr*^, DAHP synthase with feedback inhibition resistance; *aspC*, aspartate aminotransferase; *ilvE*, branched chain amino acid aminotransferase; *pheA,* chorismate mutase/prephenate dehydratase; *pheA* ^*fbr*^, chorismate mutase/prephenate dehydratase with feedback inhibition resistance; *tdiD*, l-tryptophan:phenylpyruvate aminotransferase; *trpE*, anthranilate synthase; *tyrB*, aromatic amino acid aminotransferase; *tyrA*, chorismate mutase/prephenate dehydrogenase

Equal molar amounts of Trp and PPA are needed for IPA biosynthesis through TdiD. The availability of both substrates is crucial for achieving the maximum production of IPA. Although an endogenous metabolite, native PPA might exist at a trace quantity. Thus, the insufficiency of PPA could be a bottleneck of the IPA biosynthesis in WTRCD strain. In order to investigate this possibility, sodium PPA (1.86 g/L, 10 mM) was added together with 0.5 mM IPTG at the same time to the medium containing Trp (2 g/L, 10 mM). The growth rate, substrate, and products concentrations were constantly monitored. As shown in Fig. 2, after PPA addition, cell growth of the WTRCD strain was hindered at the initial stage, although the final cell density was similar to that of the control. IPA production increased quickly from 0 to 5 h and reached 23.52 ± 3.71 mg/L at 5 h, which was approximately 3 times higher than that in the WTRCD strain without PPA supplementation. During the 5–20 h incubation time, IPA production of the WTRCD strain with PPA was enhanced 2- to 3-fold compared to the strain without PPA. However, the advantage in production disappeared after 20 h and the final IPA amount produced by WTRCD with PPA was only slightly higher than that of the strain without PPA (Fig. 2b). This decline might due to the rapid reduction in PPA levels over time. The relative concentration of PPA dropped from 100 to 25.27% in 10 h, remained 3.22% at 15 h, and was undetectable after 20 h (Fig. 2c). The maximum amount of Phe was observed at 20 h with a concentration of 0.86 ± 0.06 g/L (5.21 mM). This suggested that only 52.10% of PPA had been subjected to transamination, and the rest could undergo the degeneration process resulting from the instability of PPA. Meanwhile, 24.07 mg/L (0.12 mM) IPA was produced at 20 h through TdiD, which was correspondent to only 0.12 mM PPA consumption. Therefore, a large majority of PPA was transformed into Phe by other inherent AATs, such as AspC, TyrB and IlvE [24, 32] (Fig. 3).

The fermentation results observed during the initial 20 h indicate that the supply of PPA could efficiently enhance IPA production (Fig. 2). However, the growth impairment and instability of PPA make it inappropriate to be as an exogenous supplement as is typically done with Trp. Furthermore, it is necessary to prevent PPA from being utilized by other AATs. Therefore, augmentation of the availability of PPA and blocking the PPA-consumption branch pathways should be the next steps.

### Increased PPA pool for IPA production

The PPA formation pathway is part of the shikimate pathway for aromatic amino acid synthesis in *E. coli* (Fig. 3). Eliminating the branch pathways and side reactions were the first considerations for improving the supply of PPA. Chorismate (CHA) is the common intermediate of Phe, tyrosine (Tyr), and Trp biosynthesis. The Tyr pathway can be disrupted via deletion of *tyrA*. However, the Trp pathway is retained in this case since Trp is also a substrate for IPA biosynthesis. In addition, AspC, TyrB, and IlvE (branched chain amino acid aminotransferase), which are responsible for the conversion of PPA into Phe, should be inactivated. According to a previous study [32], double deletion of *aspC* and *tyrB* can effectively improve PPA concentration and enhance the downstream product production. Furthermore, knockout of *ilvE* was shown to reduce Phe production, but had little effect on PPA or the downstream product, and resulted in a multi-auxotrophic strain. Therefore, the *tyrA*, *tyrB,* and *aspC* mutants [24] were used for IPA biosynthesis in this study to limit carbon flux diversion away from the heterogonous pathway (Fig. 3).

**Fig. 4:**
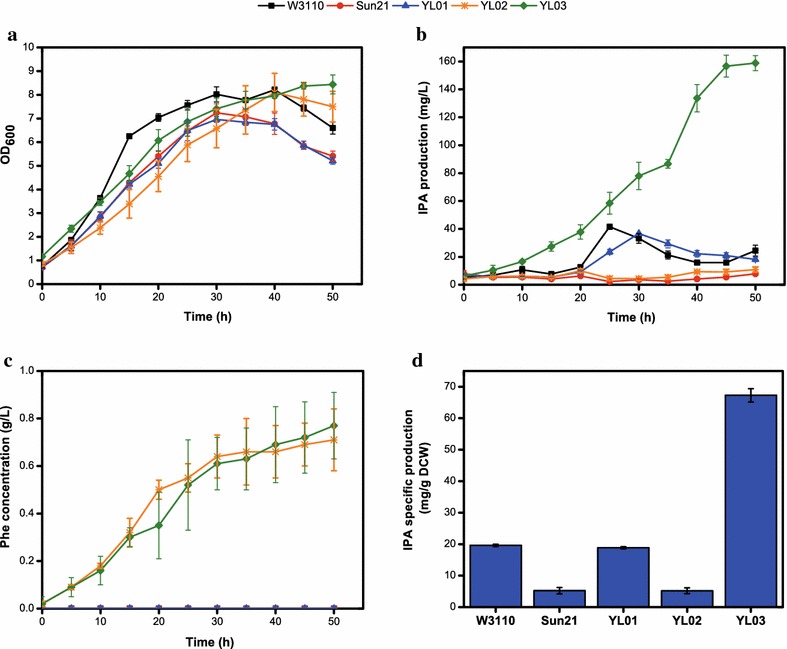
Culture profiles of W3110, Sun21, YL01, YL02, and YL03 strains. **a** Growth curve; **b** IPA production; **c** Phe concentration; **d** IPA specific production. IPA specific production here refers to the best IPA production obtained during cultivation, except for 0 h, divided by the biomass at the same time point. The time-points for IPA specific production of the various strains were dissimilar: W3110 was 25 h, YL01 was 30 h, and Sun21, YL02, and YL03 were 50 h

The *tyrA*, *tyrB* and *aspC* triple deletion strain Sun21 [24] generally loses IPA-producing capacity (Fig. 4b). This result confirms that TyrB and AspC are the major AATs responsible for basal IPA production. Then, pTRCD was introduced into the mutant strain, named YL01. Both the Sun21 and YL01 strains exhibited growth inhibition, resulting in 88.26 and 84.78% max cell density relative to the W3110 strain, respectively (Fig. 4a). This diminished cell growth can be attributed to the three inactivated genes. Unlike the WTRCD strain in which TdiD along with the native AATs work together for IPA synthesis, TdiD serves as the only enzyme for IPA production in YL01. As shown in Fig. 4b, the W3110 strain accumulated higher IPA production in the first 25 h, while the YL01 strain surpassed it in the subsequent 30–45 h of fermentation time. By 30 h, the YL01 strain accumulated 36.72 ± 0 mg/L (18.86 ± 0.32 mg/g DCW) IPA through the transaminase activity of TdiD, which was 88.40% IPA production and 96.18% IPA specific production relative to that of the W3110 strain at 25 h. Therefore, the reconstitution of *tdiD* ^*co*^ in the mutant strain Sun21 largely regained Trp transamination ability and restored the basal IPA levels of the wild-type strain. The producing capacity exhibited by the YL01 strain confirms the promising role of TdiD in IPA biosynthesis.

In order to elevate the carbon flux in the PPA synthetic pathway and thus effectively enhance the supply of precursor, overexpression of the feedback resistance of DAHP (3-deoxy-d-arabino-heptulosonate-7-phosphate) synthase (*aroF* ^*fbr*^) and chorismate mutase/prephenate dehydratase (*pheA* ^*fbr*^) are common strategies [24, 33]. The strains harboring the *aroF* ^*fbr*^ and *pheA* ^*fbr*^ expression plasmid pSUFAQ [24] in Sun21 and YL01 were YL02 and YL03, respectively (Table 1). As illustrated in Fig. 4c, large amounts of Phe were accumulated in the YL02 and YL03 strains, indicating that the carbon flux had been redirected to the Phe pathway. Since PPA is a transient metabolite and can be rapidly converted into Phe by TdiD and the residual IlvE, the quantity of Phe indicated the existence of a sufficient PPA pool. However, the YL02 and YL03 strains showed significant growth delay. This negative impact might be caused by the carbon flux decrease in the TCA cycle [34]. In spite of this, the maximum cell density attained by YL02 and YL03 were similar to that of W3110. YL02 produced negligible levels of IPA as Sun21 since the deficiency of AATs almost abolished IPA formation in both strains (Fig. 4b). With the reinforcement of linear flux from glucose to PPA, YL03 had the highest IPA production. The max IPA level was 158.85 ± 5.36 mg/L at 50 h, which is approximately 3.82-fold higher than that of the wild-type W3110. Moreover, YL03 had a max IPA specific production of 67.27 ± 2.11 mg/g DCW, corresponding to a 3.43-fold improvement compared to W3110 (Fig. 4d). As shown in Fig. 4b, the IPA production of YL03 increased continually and reached plateaus after 45 h. Compared to the IPA production profile of YL01 strain (parent strain of YL03, without pSUFAQ), this demonstrated that slowly reduced IPA concentration in YL01 after 30 h was due to the inadequate of PPA, not the consumption of IPA and the repressed enzyme expression as in W3110.

In previous reports, engineered PPA synthetic pathways have been expanded to obtain a lot of valuable products [24, 32, 33, 35]. In this study, the PPA pathway was engineered for enhanced IPA biosynthesis. The stimulation of IPA production accompanied by a certain amount of Phe production renders the IPA producing strain a multi-use platform in the future.

### Optimization of *tdiD*^*co*^ expression levels

As the IPA production shown above, pTRCD in YL01 can only compensate for the loss of the two major multispecific native AATs for IPA formation. This suggests that the expression of *tdiD* ^*co*^ could be a potentially regulatory element in IPA biosynthesis.

For fine-tuning gene expression, a constitutive system is better than an inducible conditional system [36]. Constitutive expression of *tdiD* ^*co*^ can isolate the external effect and sustain amino transfer function throughout cultivation. A series of constitutive promoters with a wide range of strength are likely involved in the regulation. The RecA promoter is a native *E. coli* promoter with strong intensity that is repressed by LexA under normal conditions [37, 38]. By abolishing the LexA binding site and mutagenesis, Oxford Genetics Ltd. (UK) constructed a series of constitutive promoters based on RecA. Among them, OXB20 is the most efficient promoter and OXBP1 is the weakest promoter. With increasing strength, OXB12, OXB14, OXB16, OXB18, and OXB20 cover a wide range of weak, moderate, and powerful promoters. These five constitutive promoters were applied to the *tdiD* ^*co*^ fine tune expression strategy.

Another considerable factor accounting for the modulation of plasmid gene expression is the copy number. The absence of ROM coding region leads to a 2- to 3-fold increase in plasmid copy number. In addition, a mutation 108A (108C) in the pBR322 origin could contribute to a 6- to 8-fold increase in copy number [39, 40]. Based on the previous study [40], conducting *rom* deletion, site-directed mutagenesis, and the combination of the two methods resulted in three high-copy-number vectors pDRD, pMOD, and pDMD (Tables 1, 2).

**Table 2:** The strains used in fine-tuning *tdiD* ^*co*^ expression

Strain	*tdiD* ^*co*^ expression plasmid	Promoter of *tdiD* ^*co*^	Relative copy number of *tdiD* ^*co*^ expression plasmid
W3110		–	–
YL03	pTRCD	Trc	1^a^
YL04	p12D	OXB12	1^a^
YL05	p14D	OXB14	1^a^
YL06	p16D	OXB16	1^a^
YL07	p18D	OXB18	1^a^
YL08	p20D	OXB20	1^a^
YL09	pDRD	OXB20	2–3 [40]^b^
YL10	pMOD	OXB20	6–8 [40]^b^
YL11	pDMD	OXB20	Approximately 16–24 [40]^b^

^a^Copy number of pTRCD with the pBR322 origin and *rom* was identified as 1, p12D, p14D, p16D, p18D, p20D have the same copy number as pTRCD

^b^pDRD, pMOD, and pDMD in YL09, YL10, and YL11, respectively had increased copy numbers

**Fig. 5:**
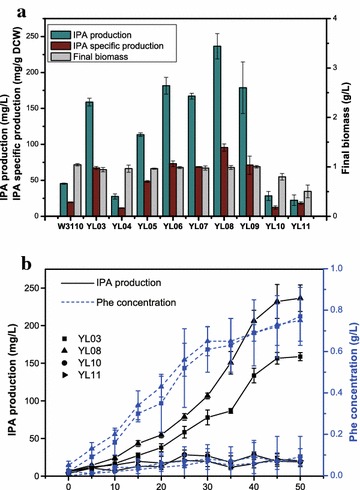
**a** IPA production, IPA specific production and the final biomass of W3110 and YL03–YL11 strains. **b** IPA production and Phe concentration of YL03, YL08, YL10, and YL11 strains during cultivation

Accordingly, eight plasmids were constructed. p12D, p14D, p16D, p18D, and p20D each contained five different constitutive promoters (Table 1). pDRD, pMOD, and pDMD had the same OXB20 promoter, but differed in plasmid copy number (Tables 1, 2). Theoretically, these eight plasmids represent the varying *tdiD* ^*co*^ expression levels. These plasmids were co-transformed into Sun21 with pSUFAQ to create the YL04–YL11 strains. To explore the effect of different levels of *tdiD* ^*co*^ expression on growth, the biomass of these producing strains in the final stage of fermentation were examined (Fig. 5a). Compared to YL03, the YL04–YL09 strains harboring p12D–p20D and pDRD, respectively (Tables 1, 2) had no apparent influence on growth and a slight increase in final biomass was observed. In contrast, YL10 showed minor growth inhibition and YL11 suffered severe growth delay; the final dry cell weight of YL11 was only about 50% of the other strains. This growth impairment might result from the metabolic burden imposed by the excessively high copy number.

For the YL04–YL08 strains, the increase in IPA production generally correlated with the improvement of promoter strength except for YL06 and YL07 (Fig. 5a). Remarkably, YL06-YL08 exhibited elevated IPA levels compared to YL03. The production of IPA in YL06 reached 181.54 ± 11.67 mg/L and YL07 reached 167.1 ± 4.08 mg/L, which increased 1.14 and 1.05 fold, respectively compared to YL03 (Fig. 5a). Moreover, YL08 showed the best IPA production performance with IPA levels of 236.42 ± 17.66 mg/L, a nearly 1.5-fold enhancement compared to YL03. In addition, the ascensive tendency was maintained throughout cultivation (Fig. 5b). Constitutive expression strategies have been widely applied in metabolic engineering to improve the accumulation of target products [41, 42], and in some cases stronger constitutive promoter could bring a better production [43]. These results, together with the previous reports, demonstrate that the constitutive expression and gradual increase of promoter strength promote the production. In contrast, the strains with a high-copy-number plasmid did not produce similar beneficial effects. IPA production in YL09 did not significantly improve. Moreover, subsequent enhancement of plasmid copy number was adverse to IPA accumulation. YL10 and YL11 exhibited sharply reduced IPA levels when a maximum concentration of 28.33 ± 6.32 mg/L (YL10) and 22.42 ± 7.27 mg/L (YL11) was obtained. This dramatic decreased IPA production was even lower than that in the wild-type W3110: only 62.23 and 49.25% of that in W3110, respectively. Compared to pTRCD in YL01, which was capable of regaining a similar production profile to that of W3110, pMOD and pDMD in YL10 and YL11, respectively, demonstrated diminished TdiD value. These results are consistent with a previous study [44] showing that the performance of enzyme encoded in plasmid did not improve as the plasmid copy number increased. Furthermore, the extremely high plasmid copy number resulted in loss of enzyme activity [33]. In addition, in YL10 and YL11, the substantial reduction of IPA production was accompanied by a decrease in Phe concentration (Fig. 5b). A possible explanation for this may be the increased expression of *tdiD* ^*co*^ induced high metabolic stress in *E. coli* and disturbed the expression of pSUFAQ. Despite the same low IPA levels, the mechanism for considerably reduced IPA production in YL04 was essentially different from that in YL10 and YL11. The insufficient *tdiD* ^*co*^ expression due to the weak promoter strength of OXB12 was responsible for the loss of IPA production in YL04. The above findings demonstrate that a modest increase in *tdiD* ^*co*^ expression benefited IPA biosynthesis.

Until now, few researches have focused on the direct production of IPA. In a previous report, 200 mg IPA was obtained from 500 mg Trp through immobilized enzymes in a continuous flow reactor [45]. And another research achieved preeminent IPA production through a novel oxidase when supplied with an enormous amount of Trp, but extra catalase was still necessary for reaching the highest production [17]. At the shake flask fermentation under the condition of 2 g/L Trp, YL08 enabled an effectively enhanced IPA production (236.42 ± 17.66 mg/L), and the IPA specific production was 95.69 ± 4.91 mg/g DCW, representing a 5.19- and 4.88-fold increase over W3110, respectively. However, the max IPA production still has not reached a satisfactory level. Based on the final IPA and Phe concentrations, even in YL08, only 25.62% of the PPA pool was employed by TdiD. Despite the existence of IlvE as a competitor, the primary reason might be the insufficient TdiD catalytic activity. Therefore, further research should focus on engineering the enzymatic activity of TdiD.

### *tnaA* influence on IPA synthesis

Tryptophanase catalyzes the decomposition of Trp into indole, ammonium, and pyruvate [46]. Deletion of the *tnaA* gene has been a common strategy for improved Trp biosynthesis production in *E. coli* [47, 48]. The expression of *tnaA* is induced by surplus Trp [49], and is inhibited by catabolite repression [50]. However, analysis of IPA biosynthesis in *E. coli* BL21 showed that deletion of *tnaA* did not have a positive effect on IPA production but resulted in a 7% reduction (Additional file 1: Figure S1).

**Fig. 6:**
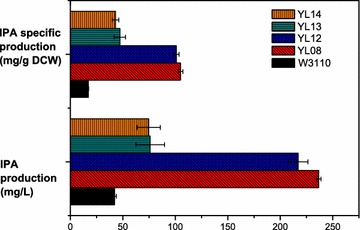
IPA production and specific production of W3110, YL08, and YL12–YL14 strains

**Fig. 7:**
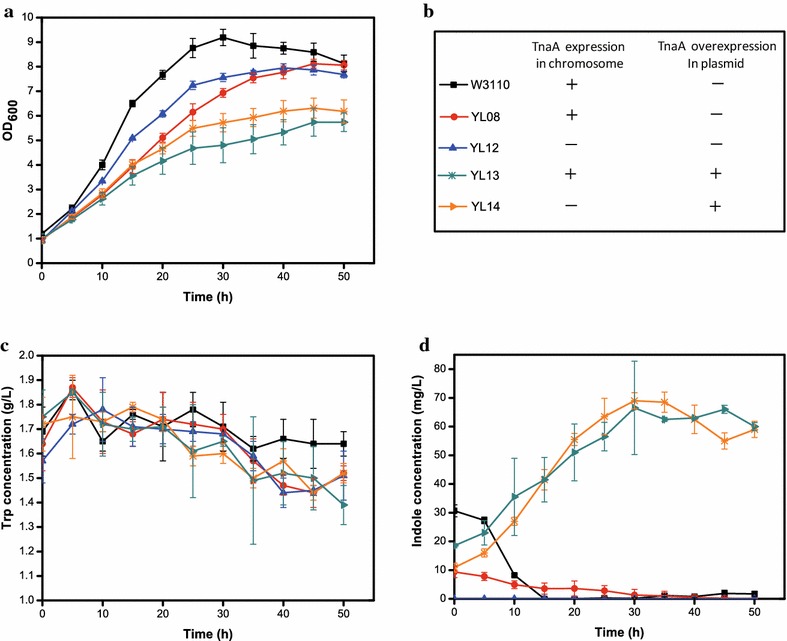
Culture profiles of W3110, YL08, and YL12–YL14 strains. **a** Growth curve; **b** symbols and schematic representation of *tnaA* expression; **c** Trp concentration; **d** indole concentration

To fully explore the potential role of *tnaA* in IPA production, a *tnaA* knockout strain of Sun21 was constructed and named Zhu01. p20D and pSUFAQ were simultaneously expressed in Zhu01 to create the YL12 strain. Furthermore, a complete *tnaA* expression cassette (including Trc promoter and T7 terminator) was inserted into p20D, resulting in pTAD. pTAD was co-transformed with pSUFAQ in either Sun21 or Zhu01 to generate YL13 or YL14, respectively. The four strains represent different *tnaA* expression levels (Fig. 7b). IPA production, Trp and indole concentration as well as the growth rate of each strain were evaluated (Figs. 6, 7). Deletion of *tnaA* in YL12 strain had no significant impact on IPA production, and only led to slight decrease of IPA production and specific production (91.69 and 96.24% of YL08, respectively) (Fig. 6). On the other hand, the IPA production of *tnaA* overexpression strains (YL13 and YL14) sharply declined, which were as low as 32.16 and 31.55% of YL08, respectively (Fig. 6). There were no obvious differences in Trp concentrations between the four strains throughout the cultivation (Fig. 7c). Therefore, the fluctuation in IPA production was not due to the Trp availability.

Overexpression of *tnaA* in YL13 and YL14 strains enabled a significant increase in indole production, while the deletion of *tnaA* in YL12 led to the complete loss of the indole-producing ability (Fig. 7d). The maximum indole levels of YL13 and YL14 were both around 70 mg/L (Fig. 7d). Although up to 357 mg/L indole was required for serious inhibition of growth caused by its ionophore action [51], a slight growth obstruction could still be observed in YL13 and YL14 (Fig. 7a). Despite the decomposition of Trp for the certain indole production, YL13 and YL14 strains shared similar Trp concentration to YL08 strain. Therefore, more carbon flux partitioning at the CHA nod of YL13 and YL14 strains might be governed towards the Trp biosynthesis pathway, such that the PPA pool is diminished. The assumption is supported by the Phe production in YL13 and YL14 that decreased to only 70.78 and 65.17% of YL08 relatively. This could explain why YL13 and YL14 both demonstrated reduced IPA production. However, the reason for the tenuous loss of IPA production as a result of *tnaA* deletion is still unclear. Possible explanations might include the effect on TdiD enzyme activity and disturbance of Trp uptake.

### IPA production with various concentrations of Trp supplement

**Fig. 8:**
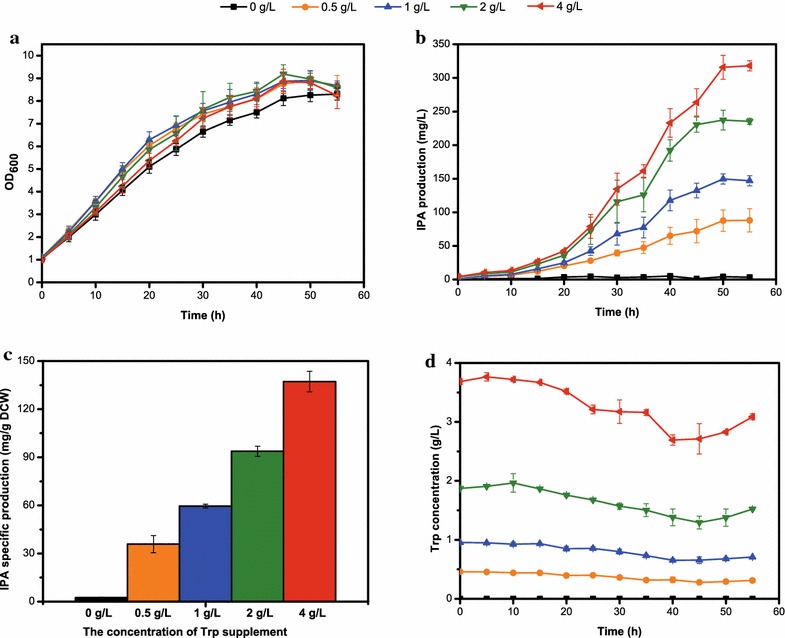
Culture profiles of YL08 strain supplied with Trp in various concentrations. **a** Growth curve; **b** IPA production; **c** IPA specific production; **d** Trp concentration

Even in the best performing strain YL08, certain amount of Trp was remained at the end of fermentation (Fig. 7c). In order to figure out whether or not there is feedback effect of Trp, other than 2 g/L Trp, 0 g/L, 0.5 g/L, 1 g/L and 4 g/L Trp were also used to investigate their effect on IPA biosynthesis of YL08 strain. The cell growth, IPA production and Trp levels of YL08 strain were determined (Fig. 8). The growth of YL08 in the mediums containing various concentrations of Trp were similar and better than in the media without Trp supplement (Fig. 8a). Without Trp (0 g/L), the IPA production was negligible and the IPA production gradually enhanced as the exogenous Trp concentration increased (Fig. 8b). YL08 strain supplied with 4 g/L Trp has the maximum IPA production (318.09 ± 7.56 mg/L), which was 1.34-fold higher than the IPA production with 2 g/L Trp supplement. Moreover, IPA productivity also elevated as the Trp concentration increased. The highest IPA productivity was obtained with 4 g/L Trp supplement and reached 6.32 ± 0.36 mg/L/h. Since substrate inhibition usually leads to the severely decreased cell mass and product formation rate as the substrate concentration increases [52, 53], here up to 4 g/L Trp showed no obvious substrate feedback for IPA production.

Provided with 4 g/L Trp, YL08 strain achieved significant IPA specific production (137.24 ± 6.37 mg/g DCW) (Fig. 8c). However, when the Trp concentration increased from 0.5 to 1 g/L, then 2 and 4 g/L, the corresponding IPA specific production only had a 1.66-, 1.57- and 1.46-fold increase respectively (Fig. 8c). As discussed above, IlvE could be a strong competitor for the PPA pool. Thus the limited PPA concentration for IPA production might be the main reason for the disproportionate improvement of IPA specific production as the Trp concentration increased. Figure 8d showed the Trp concentration profile of the YL08 strain with different Trp supplement. From 0 to 40 h, the Trp levels of the culture decreased slowly, but then started to increase after 45 h fermentation time. Possible explanation might be the endogenous Trp biosynthesis through the shikimate pathway.

Consequently, future attempt to increase IPA biosynthesis production should be addressed from improvement of enzyme activity and substrate affinity of TdiD. Meanwhile, instead of exogenous supplement, Trp biosynthesis from glucose through inherent pathway should be performed as well. Strategies [54] for further shikimate pathway engineering could be implemented for simultaneous sufficient supply of both Trp and PPA without artificial addition.

## Conclusions

In this study, a series of strains for IPA biosynthesis were established. The YL03 strain exhibited a 158.85 ± 5.36 mg/L IPA production, representing a 3.82-fold increase over that in the wild-type W3110. Furthermore, YL08 showed the best exaltation with IPA titers and was capable of producing 236.42 ± 17.66 mg/L IPA in flask cultivation. This represents an excellent starting point as a new avenue for future IPA biosynthesis via TdiD. Construction of the YL08 strain involved engineering the biosynthetic pathway and optimizing *tdiD* ^*co*^ expression levels. Following study aimed at identifying the role of *tnaA* in IPA biosynthesis. In addition to the consistent unfavorable influence, there was some discrepancy between the effects of *tnaA* deletion and its overexpression on IPA production. Finally, the IPA production with various concentrations of Trp supplement indicated that higher Trp concentration would benefit the IPA production. Overall, this study provides detailed evidence and serves as a useful resource for the future research.

## Methods

### Strains and chemicals

The strains and plasmids used in this study are listed in Table 1. *E. coli* strain DH5α was purchased from Transgene Bio (Transgene Biotechnology Co. Ltd. Beijing, China) for gene cloning. For IPA biosynthesis, *E. coli* strains W3110, mutant Sun21, and plasmid pSUFAQ were kindly donated by Professor Sheng Yang (Key Laboratory of Synthetic Biology, Institute of Plant Physiology and Ecology, Shanghai Institutes for Biological Sciences, Chinese Academy of Sciences, Shanghai, China). Strains were maintained as glycerol stocks at −80 °C. The IPA sample was purchased from Sigma. All the other chemicals were purchased from Sangon Bio (Sangon Biotechnology Co. Ltd. Shanghai, China). Restriction endonucleases, DNA polymerases, and T4 DNA ligase were purchased from Takara Bio (Takara Biotechnology Dalian Co. Ltd., China) or Sangon Bio.

### Culture media

Lysogeny broth (LB) medium containing 10 g/L tryptone, 5 g/L yeast extract, and 10 g/L NaCl was used for *E. coli* cultivation. For IPA biosynthesis, *E. coli* strains were cultured in A medium composed of the following components (1 L): 20 g glucose, 2 g (NH_4_)_2_SO_4_, 13.6 g KH_2_PO_4_, 0.2 g MgSO_4_·7H_2_O, 7.5 × 10^−3^ g CaCl_2_, 5 × 10^−4^ g FeSO_4_·7H_2_O, 2 g Trp, 0.2 g Tyr, and 3 g aspartic acid. The pH was adjusted to 7 by the addition of NaOH. If required, antibiotics were added at appropriate concentrations: kanamycin 50 g/L, ampicillin 100 g/L, or chloramphenicol 35 g/L.

### Plasmid construction

Gene cloning was conducted according to standard protocols [55]. Gene splicing and site-directed mutagenesis were carried out according to a previously published PCR-mediated technique [56]. PCR primers, *tdiD* ^*co*^ and OXB18 promoter were synthesized by Sangon Bio. The sequence of *tdiD* ^*co*^ was deposited in GenBank under the accession number KX594383.

All primers used in this study are listed in Additional file 1: Table S1. To construct pTRCD, PCR-amplified *tdiD* ^*co*^ from pUC57-D using the primers tdiD^co^-F/tdiD^co^-R was digested with *Nde*I*/Not*I and cloned into the *Nde*I*/Not*I site of the *tdiD* gene removed pET24b-*tdiD*, resulting in pT7D. The *tdiD* ^*co*^ in pT7D completely replaced the *tdiD* in pET24b-*tdiD*. Next, the Trc promoter amplified from pBJEI-6409 with the primers Trc-F-1/Trc-R-1 was substituted for the T7 promoter at the *Bgl*II*/Nde*I site of pT7D to generate pTRCD. The promoter changes mentioned below were all performed using the same approach through the *Bgl*II*/Nde*I site. The OXB18 promoter was amplified from pUC57-18 using the primers 18-F/18-R and substituted with the Trc promoter in pTRCD to obtain a new plasmid named p18D. OXB12, OXB14, OXB16, and OXB20 promoters are mutants of OXB18 promoter. To generate the OXB14 promoter, two PCR products were amplified from the OXB18 promoter in p18D with the primers 18-F/14-1 and 14-2/18-R, respectively. Then the two overlapping fragments were fused through overlap extension PCR to obtain full length OXB14 using the primers 18-F/18-R. The OXB14 promoter was subsequently cloned into p18D, and replaced the OXB18 promoter to yield p14D. A similar method was used to construct p20D. Using OXB20 in p20D as the template, the promoters OXB12 and OXB16 were also constructed with different combinations of corresponding primers with 18-F/18-R. They were then used to obtain plasmids p12D and p16D, respectively. To construct the pDRD plasmid, the linear plasmid backbone was amplified from p20D using the primers DR-F/DF-R to remove *rom* and ligated together by the added restriction site *Sal*I. To generate pMOD, the mutational pBR322 origin was obtained though overlap PCR with primers (MO-1 to MO-4) containing the mutation within the origin region [108A (108C)]. Then, the mutated pBR322 origin was ligated with the plasmid backbone amplified from p20D using primers MO-5/MO-6, by the added restriction site *Nco*I. Deletion of *rom* in pMOD was accomplished by the same means used for pDRD with the same primers DR-F/DR-R to produce pDMD. Construction of the *tnaA* expression cassette consisted of amplification of the Trc promoter from pTRCD using the Trc-F-2/Trc-R-2 primers, amplification of *tnaA* from W3110 with primers tnaA-F/tnaA-R, amplification of the T7 terminator from pTRCD with primers Ter-T7-F/Ter-T7-R, and the fusion of the three fragments through overlap extension PCR. The resulting hybrid sequence amplified from the above three products with primers Trc-F-2/Ter-T7-R, which contained the *tnaA* gene flanked by the Trc promoter and T7 terminator, was incorporated into p20D through the single restriction site *Bgl*II to generate pTAD.

### Construction of *tnaA* deletion mutant

The deletion of *tnaA* was achieved using a one-step inaction method [25]. Generally, the PCR fragment used to mediate gene replacement for *tnaA* was amplified from pKD13 using the primers p1-tnaA/p4-tnaA and electroporated into the competent Sun21 strain harboring pKD46. After confirmation of the replacement by PCR with the primers kan-up/kan-down, kanamycin-resistance (Km^R^) marker was removed by pCP20 to generate the finally *tnaA* deletion strain, Zhu01. Verification of the disruption was conducted by PCR with primers up-tnaA/down-tnaA and DNA sequencing

### Shake flask fermentation for IPA biosynthesis in *E. coli*

Overnight LB medium culture (2 mL) was inoculated in 250 mL Erlenmeyer flasks containing 50 mL of A medium. The cultures were first maintained in a shaker at 37 °C and 250 rpm. When the OD_600_ reached around 0.7–1.0, a final concentration of 0.5 mM IPTG was added. For the PPA feeding experiment, 1.86 g/L sodium PPA was supplemented at the same time of IPTG. The temperature was then reduced to 25 °C for enhanced soluble expression of proteins. In order to keep the stability of IPA, the cultures were kept in dark throughout the growth period. Every 5 h, 0.5 mL samples were taken out. A portion of the samples were used for cell density tests, and the rest were centrifuged at 4 °C and 10,000 rpm for 10 min. The supernatants were then stored at −20 °C for subsequent IPA and HPLC detection. All the experiments in this report were conducted in three independent replicates.

### Determination of bacteria biomass

In order to monitor cell growth, the optical density of the culture was determined by measuring the absorbance at OD_600_ using an UV2300 UV–Vis (ultraviolet–visible) spectrophotometer (Techcomp, Shanghai, China) after an appropriate dilution. For the dry cell weight (DCW) measurement, 10 mL of culture broth was collected by centrifugation at 10,000×*g* for 10 min in a pre-weighed tube. Harvested cell was washed twice with deionized water, and then dried at 90 °C to a constant weight. Dry cell weight was calculated using the formula obtained in this work: DCW (g/L) = 0.28 × OD_600_.

### IPA measurement

Salkowski reagent has been widely used to estimate IAA production through a colorimetric method [57–59]. The measurement results obtained with the Salkowski reagent share a similar dynamic tendency to HPLC results [60]. However, an accurate analysis [61] reveals that, besides IAA, Salkowski reagent can also react with IPA, and indole acetamide (IAM). When used in a reaction with IPA, the Salkowski reagent can provide precise measurements of AAT activity, and the accuracy of the method has been demonstrated [62]. In this study, without IAA and IAM in *E. coli*, the Salkowski reagent can be a suitable way for IPA quantification.

Among the three formulations of the Salkowski reagent, the PC technique is the most sensitive and specific [61]. Trp barely responds to the PC reagent, but its appearance improves the sensitivity towards IPA. When tested, the concentration of Trp in all samples and the standard were adjusted to 1 g/L. The supernatant was diluted 20 or 40 times with culture medium and water, then 0.5 mL of the sample was mixed with 0.5 mL of the PC reagent, which consists of 12 g/L FeCl_3_ and 7.9 M H_2_SO_4_. After 30 min incubation in the dark at room temperature, absorbance was read at 530 nm using the UV2300 UV–Vis spectrophotometer. IPA production was calculated from the standard calibration curve.

### HPLC quantification

The concentrations of Trp, PPA, Phe, and indole were determined using an Agilent 1260 series HPLC (Agilent Technologies, USA) equipped with a UV–Vis diode array detector. A ZORBAX SB-18 column (5 μm, 4.6 × 150 mm) maintained at 35 °C was used for sample separation. The mobile phase consisted of 0.03% KH_2_PO_4_ water (solvent A) and methanol (solvent B) with a 1 mL/min flow rate. After injection of 10 μL diluted sample, the gradient was proceeded as follows: 20% B (0–2 min), 60% B (4–8 min), 100% B (14–19 min), and 20% B (20–25 min). The wavelength used to detect Phe and PPA was 215 nm, whereas the wavelength used to detect Trp and indole was 280 nm.

## Additional file

**Additional file 1: Figure S1.** The IPA biosynthesis in BL21 strain. BLA is the *tnaA* knockout strain of BL21. The IPA level of BL21 + pT7D represents the relative 100% production. Similarly, the IPA specific production (mg/g DCW) of BL21+ pT7D represents the relative 100% specific production. **Table S1.** Primers used in this study.

## Abbreviations

AAT: amino acid aminotransferase
ANTA: anthranilate
CHA: chorismate
DAHP: 3-deoxy-d-arabino-heptulosonate-7-phosphate
E4P: erythrose 4-phosphate
HPP: 4-hydroxyphenylpyruvate
IAA: indole-3-acetic acid
IAM: indoleacetamide
IPA: indole pyruvic acid
PEP: phosphoenolpyruvate
Phe: phenylalanine
PPA: phenylpyruvate
Trp: tryptophan
Tyr: tyrosine
