# Supplementary material for: Metabolic engineering of indole pyruvic acid biosynthesis in Escherichia coli with tdiD
Source: Microb Cell Fact. 2017 Jan 3;16:2. doi: 10.1186/s12934-016-0620-6 (PMC5209907; doi:10.1186/s12934-016-0620-6)
Supplement: Supplementary file 1 — Additional file 1: Figure S1. The IPA biosynthesis in BL21 strain. BLA is the tnaA knockout strain of BL21. The IPA level of BL21 + pT7D represents the relative 100% production. Similarly, the IPA specific production (mg/g DCW) of BL21+ pT7D represents the relative 100% specific production. Table S1. Primers used in this study. [file 12934_2016_620_MOESM1_ESM.docx]

**Additional file 1**

**
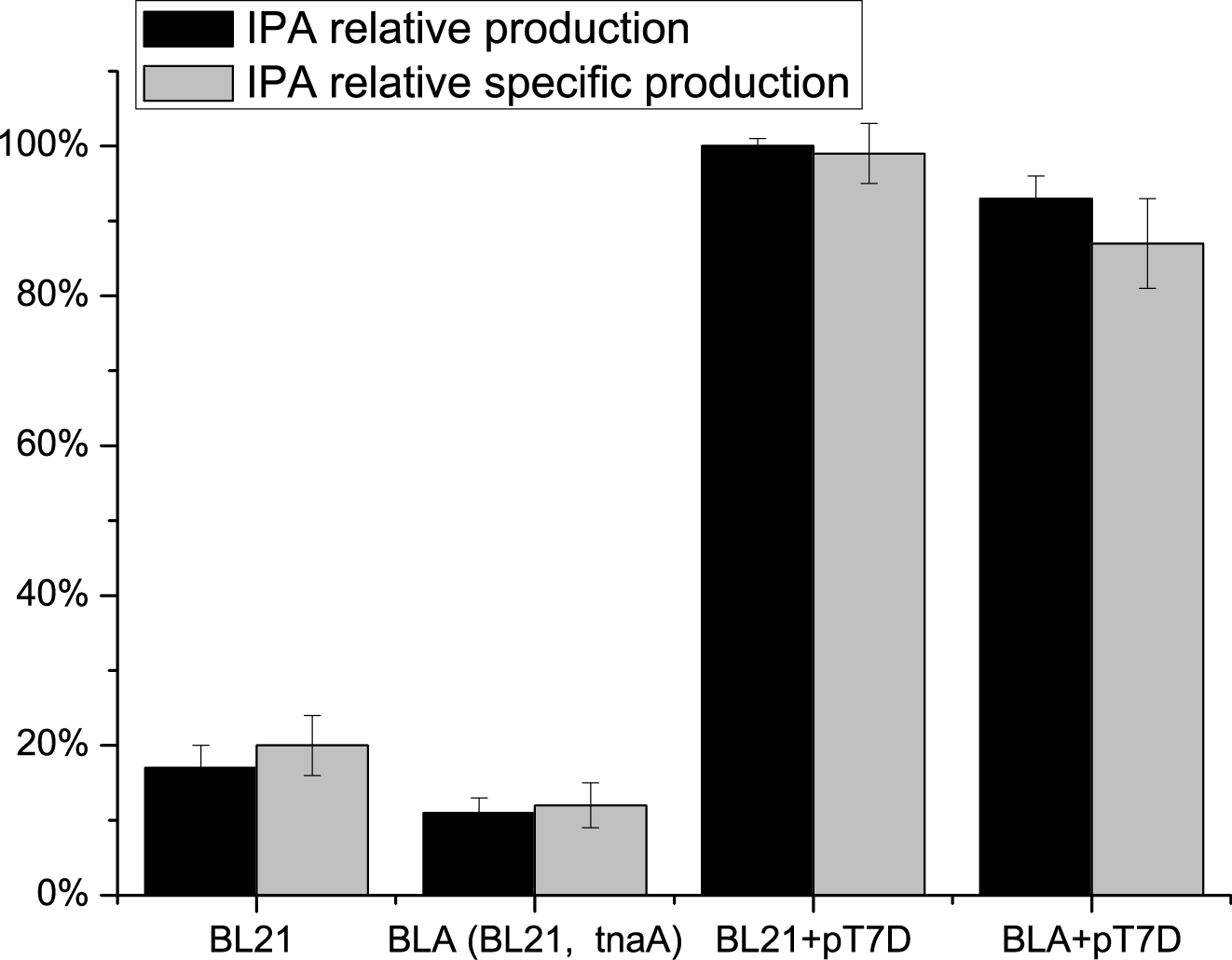
**

**Figure S1.** **The IPA biosynthesis in BL21 strain**. BLA is the *tnaA* knockout strain of BL21. The IPA level of BL21 + pT7D represents the relative 100% production. Similarly, the IPA specific production (mg/g DCW) of BL21+ pT7D represents the relative 100% specific production.

**Table S1.** Primers used in this study

| Primers | Sequence(5′-3′, restriction site underlined) |
| --- | --- |
| tdiD^co^-F | CAAGTTACATATGGGTTCTATTGGTGCAAACAACG |
| tdiD^co^-R | CAAGTTATGCGGCCGCAGAATGGCATGCGCAACGATC |
| Trc-F-1 | CAAGTTAAGATCTTTCTGAAATGAGCTGTTGACAA |
| Trc-R-1 | CAAGTTACATATGGGTTATTTCCTCCTAGATCCTG |
| 18-F | GGAAGATCTAACTGCTGATCGAGTGTAGC |
| 18-R | GGAATTCCATATGGGGTACCTCCTTTGAATTCG |
| 14-1 | TGTGGACCTTATCGAGCGATCGAGCA |
| 14-2 | TGCTCGATCGCTCGATAAGGTCCACA |
| 20-1 | TATAGCAGCTACGTGGACCTTATCAAGCGATCGAG |
| 20-2 | CTCGATCGCTTGATAAGGTCCACGTAGCTGCTATA |
| 12-1 | ACGTGGACCTTACCAAGCGATCGAGC |
| 12-2 | GCTCGATCGCTTGGTAAGGTCCACGT |
| 16-1 | GTTGAAGCAATTGTAGCAGCTACGTG |
| 16-2 | CACGTAGCTGCTACAATTGCTTCAAC |
| DR-F | ACGCGTCGACTGATGCCTCCGTGTAAGGGGGA |
| DR-R | ACGCGTCGACCACATGCAGCTCCCGGAGACGG |
| MO-1 | CATGCCATGGCGCGTTGCTGGCGTTTTTCCAT |
| MO-2 | GATCAAGAGCTAACAACTCTTTTTC |
| MO-3 | GAAAAAGAGTTGTTAGCTCTTGATC |
| MO-4 | CATGCCATGGAGATCAAAGGATCTTCTTGAGA |
| MO-5 | CATGCCATGGGCCTTTTTACGGTTCCTGGCCT |
| MO-6 | CATGCCATGGTTTCTACGGGGTCTGACGCTCA |
| Trc-F-2 | GGAAGATCTTTCTGAAATGAGCTGTTGAC |
| Trc-R-2 | AGTTTTCCATGGTTATTTCCTCCTAGATCC |
| tnaA-F | GGAAATAACCATGGAAAACTTTAAACATCT |
| tnaA-R | CAGCCGGATCTTAAACTTCTTTAAGTTTTG |
| Ter-T7-F | AGAAGTTTAAGATCCGGCTGCTAACAAAGC |
| Ter-T7-R | GGAAGATCTATCCGGATATAGTTCCTCCT |
| P1-tnaA | ATGGAAAACTTTAAACATCTCCCTGAACCGTTCCGCATTCGTGTTATTGAGTGTAGGCTGGAGCTGCTTC |
| P4-tnaA | TTAAACTTCTTTAAGTTTTGCGGTGAAGTGACGCAATACTTTCGGTTCGTATTCCGGGGATCCGTCGACC |
| up- tnaA | GGCGGATTTTCTCCAGCTTCTGTA |
| down-tnaA | CAATACGCTGGCAACGAAAATGGC |
| kan-up | cttcggaataggaacttcaagatcccctta |
| kan-down | gggctatctggacaagggaaaacgcaagcg |
